# Prediction model for delirium in patients with cardiovascular surgery: development and validation

**DOI:** 10.1186/s13019-022-02005-3

**Published:** 2022-10-01

**Authors:** Yanghui Xu, Yunjiao Meng, Xuan Qian, Honglei Wu, Yanmei Liu, Peipei Ji, Honglin Chen

**Affiliations:** 1 Departments of Cardiovascular Surgery Intensive Care Unit, Affiliated Hospital of Nantong University, Nantong, China; 2 School of Public Health, Nantong University, No.9, Sik Yuan Road, Nantong, China

**Keywords:** Cardiovascular surgery, Delirium, Nomogram model, Discrimination, Calibration

## Abstract

**Background:**

The aim of this study was to construct a nomogram model for discriminating the risk of delirium in patients undergoing cardiovascular surgery.

**Methods:**

From January 2017 to June 2020, we collected data from 838 patients who underwent cardiovascular surgery at the Affiliated Hospital of Nantong University. Patients were randomly divided into a training set and a validation set at a 5:5 ratio. A nomogram model was established based on logistic regression. Discrimination and calibration were used to evaluate the predictive performance of the model.

**Results:**

The incidence of delirium was 48.3%. A total of 389 patients were in the modelling group, and 449 patients were in the verification group. Logistic regression analysis showed that CPB duration (OR $=$ 1.004, 95% CI: 1.001–1.008, $P=$ 0.018), postoperative serum sodium (OR $=$ 1.112, 95% CI: 1.049–1.178, $P<$ 0.001), age (OR $=$ 1.027, 95% CI: 1.006–1.048, $P=$ 0.011), and postoperative MV (OR $=$ 1.019, 95% CI: 1.008–1.030, $P<$ 0.001) were independent risk factors. The results showed that AUC$^\text {ROC}$ was 0.712 and that the 95% CI was 0.661–0.762. The Hosmer-Lemeshow goodness of fit test showed that the predicted results of the model were in good agreement with the actual situation ($\chi ^{2}=$ 6.200, $P=$ 0.625). The results of verification showed that the AUC$^\text {ROC}$ was 0.705, and the 95% CI was 0.657–0.752. The Hosmer-Lemeshow goodness of fit test results were $\chi ^{2}=$ 8.653 and $P=$ 0.372, indicating that the predictive effect of the model is good.

**Conclusions:**

The establishment of the model provides accurate and objective assessment tools for medical staff to start preventing postoperative delirium in a purposeful and focused manner when a patient enters the CSICU after surgery.

## Background

Delirium is a common complication after cardiovascular surgery. It manifests as a disturbance of consciousness and attention, accompanied by changes in cognitive function or perceptual disturbance, and it is characterised by acute onset and repeated fluctuations of the disease [1]. Postoperative delirium is especially common after cardiovascular surgery, and its incidence can be as high as 52% [2]. It is a crucial public health concern, as it is strongly associated with an increased incidence of other postoperative complications. Moreover, postoperative delirium is associated with prolonged mechanical ventilation. Delirium is also related to an increase in length of hospital stay and hospital costs [3]. A recent study identified an association between delirium and long-term cognitive dysfunction in patients. In severe cases, delirium may also increase in-hospital mortality [4] and long-term mortality after discharge [5].

Randomised controlled studies have shown that shortening the duration of delirium through clinical intervention does not effectively reduce short-term mortality [6]. Therefore, the prevention of delirium is a very important clinical research topic at present. Predictive nomograms, which have been broadly used as one of the most common statistical methods for clinical investigations, not only offer visual and mathematical advantages but also facilitate probability calculation of risk factors; they are also easier to use clinically [7]. These clinical nomograms may aid in the decision-making process as well as in the perioperative management of cardiovascular surgery patients. They can also contribute to the allocation of postoperative care resources to cardiovascular surgery intensive care units (CSICUs) and will provide administrators, physicians, and nurses with a guide to the estimated length of hospital stay. Thus, a nomogram-based clinical prediction model capable of early identification of patients at risk for delirium can prove useful both in the proper management of these patients and in the efficient allocation of healthcare resources [8, 9].

The design and writing of this research paper refers to the transparent reporting of a Multivariable Prediction Model for Individual Prognosis or Diagnosis (TRIPOD) [10, 11].

## Methods

### Patients and study design

A retrospective study was conducted on a cohort of patients who underwent cardiovascular surgery between January 2017 and November 2019. A prospective study was conducted on a cohort of patients who underwent cardiovascular surgery between December 2019 and June 2020 at the Affiliated Hospital of Nantong University. All the collected data were sorted and randomly grouped for research.

Inclusion criteria included the following: (1) patients who entered the CSICU for continued treatment after cardiovascular surgery and (2) adult patients. The exclusion criteria were as follows: (1) severe adverse events, such as cardiac arrest and/or cardiopulmonary resuscitation, that occurred during surgery; (2) history of alcohol addiction or drug abuse; (3) a previous history of mental illness; (4) perioperative neurological complications; (5) postconciousness disorder; (6) postoperative death; (7) aortic dissection interventional surgery; and (8) rehospitalization or readmittance to the CSICU. The study was approved by the institutional ethics committee.

### Diagnostic criteria for delirium

The diagnostic criteria for delirium in this study adopted the fuzzy assessment of ICU patients (CAM-ICU) designed by Professor Ely [12]. CAM-ICU is mainly diagnosed based on the following four points: (1) acute onset and fluctuating course of the disease; (2) lack of attention and concentration; (3) disordered thinking; and (4) change in consciousness. If the patient has (1) and (2), then the presence of either (3) or(4) can be diagnosed as delirium. The scale is a delirium assessment tool developed for critically ill patients, and it is recommended by the guidelines as the gold standard for delirium screening [ 3].

### Data collection content

The demographic characteristics were gender, age (years old), body mass index (BMI), blood type, and past history. The clinical characteristics were type of surgery, cardiopulmonary bypass (CPB) duration, intraoperative minimum mean arterial pressure (MAP), intraoperative blood transfusion, and postoperative mechanical ventilation (MV) duration. Finally, the laboratory test indicators were white blood cell count (WBC) (the highest or lowest value that deviated from the normal value), red blood cell-specific volume haematocrit (Hct) (the lowest value that deviated from the normal value), alanine aminotransferase (ALT) (the highest value that deviated from the normal value), total bilirubin (TBIL) (the highest value that deviated from the normal value), serum albumin (the lowest value that deviated from the normal value), serum urea nitrogen (the highest value that deviated from the normal value), serum creatinine (Cr) (the highest value that deviated from the normal value), and serum sodium (the highest or the lowest value that deviated from the normal value). Laboratory test indicators were collected within 24 h after the patient entered the CSICU after surgery. The above information could be obtained from the electronic medical records.

### Statistical analysis

(1) The generation of the training set and validation set: We use the random number generator and the approximation method to divide 838 research subjects into a training set and validation set at a ratio of 5:5 in SPSS. (2) Construction of the model: We investigated possible risk factors by univariate analysis. Continuous variables were tested for statistical significance by Student’s t test. Categorical variables were tested by the chi-square test. Then, logistic regression analysis was performed. The variables finally included in the logistic regression equation were introduced into R software (4.0.3), and the rms program package was used to establish a nomogram prediction model, with a two-sided $P<$ 0.05 indicating significance. (3) The internal evaluation of the model in the training set: we tested the calibration and discrimination of the nomogram prediction model. The discrimination was evaluated by the area under the curve (AUC). The AUC range was 0.5 $\sim$ 1.0. The closer the AUC was to 1, the better the predictive power of the model was. An AUC of 0.5 indicates that the model has no predictive power. AUC > 0.7 indicates that the model has good predictive ability [13]; the calibration was evaluated by the calibration curve and goodness-of-fit test, and $P>$ 0.20 was recognised as an acceptable calibration. (4) Evaluation of the model in the validation set: first, the established model was used to score the validation set, and then the area under the curve, the calibration curve, and the goodness-of-fit test were used to evaluate the accuracy of the model in the validation set.

## Result

A total of 838 patients were enrolled from January 2017 to June 2020. All patients were randomly divided into a training set and a validation set. Among them, 389 patients were in the training set, and almost 48.8% of patients (190) had delirium; 449 patients were in the validation set, and almost 47.9% of patients (215) had delirium. There was no significant difference in the comparison of the variables between the two groups ($P>$ 0.05). The baseline characteristics in both groups are shown in Table 1.

**Table 1:** Descriptive analysis of the training set and validation set data

Variable	Total	Training set	Validation set	$\chi ^{2}$/t	*P*
	n (%)/$\bar{x} \pm s$	n (%)/$\bar{x} \pm s$	n (%)/$\bar{x} \pm s$		
Delirium	405 (48.3)	190 (48.8)	215 (47.9)	0.077	0.782
Gender				0.061	0.804
Male	496 (59.2)	232 (59.6)	264 (58.8)		
Female	342 (40.8)	157 (40.4)	185 (41.2)		
Age	60.0 ± 11.8	59.8 ± 11.2	60.2 ± 12.2	0.477	0.634
BMI	23.9 ± 3.3	24.0 ± 3.4	23.9 ± 3.3	−0.580	0.562
Blood type				3.673	0.299
A	269 (32.1)	133 (34.2)	136 (30.3)		
B	236 (28.2)	107 (27.5)	129 (28.7)		
AB	87 (10.4)	33 (8.5)	54 (12)		
O	246 (29.4)	116 (29.8)	130 (29.0)		
Past history					
Hypertension	385 (45.9)	175 (45.0)	210 (46.8)	0.267	0.605
Diabetes	147 (17.5)	63 (16.2)	84 (18.7)	0.910	0.340
Stroke	48 (5.7)	23 (5.9)	25 (5.6)	0.046	0.830
Atrial fibrillation	190 (22.7)	92 (23.7)	98 (21.8)	0.396	0.529
Malignant tumor	35 (4.2)	15 (3.9)	20 (4.5)	0.186	0.666
Type of surgery				1.885	0.865
CABG	193 (23)	96 (24.7)	97 (21.6)		
Valve replacement or shaping	406 (48.4)	185 (47.6)	221 (49.2)		
Aortic valve replacement or shaping	45 (5.4)	18 (4.6)	27 (6.0)		
Heart tumor removal	22 (2.6)	10 (2.6)	12 (2.7)		
Congenital correction	52 (6.2)	23 (5.9)	29 (6.5)		
Two kinds of surgery and above	120 (14.3)	57 (14.7)	63 (14)		
CPB duration	102.8 ± 75.6	98.8 ± 73.3	106.2 ± 77.4	1.414	0.158
Intraoperative minimum MAP	40.4 ± 11.5	41.1 ± 11.2	39.8 ± 11.7	−1.583	0.114
Intraoperative blood transfusion	152 (18.1)	72 (18.5)	80 (17.8)	0.067	0.796
Postoperative MV	31.9 ± 57.3	33.3 ± 71.5	30.7 ± 41.4	−0.644	0.520
Postoperative WBC	13.3 ± 4.1	13.4 ± 4.3	13.3 ± 4.0	−0.649	0.516
Postoperative Hct	0.3 ± 0.04	0.31 ± 0.04	0.31 ± 0.04	0.946	0.345
Postoperative ALT	45.8 ± 59.1	48.6 ± 68.9	43.4 ± 49.0	−1.281	0.200
Postoperative TBIL	25.3 ± 13.2	25.8 ± 14.4	24.7 ± 12.0	−1.201	0.230
Postoperative serum albumin	31.6 ± 5.6	31.6 ± 5.9	31.7 ± 5.3	0.109	0.913
Postoperative serum urea nitrogen	9.5 ± 3.6	9.2 ± 3.5	9.7 ± 3.7	1.848	0.065
Postoperative Cr	102.5 ± 40.9	101.8 ± 41.7	103.2 ± 40.2	0.490	0.624
Postoperative Serum Sodium	146.1 ± 4.7	146.0 ± 4.3	146.1 ± 5.0	0.235	0.814

*BMI*, body mass index; *CPB*, Cardiopulmonary Bypass; *MAP*, mean arterial pressure; *MV*, mechanical ventilation; *WBC*, white blood cell; *Hct*, Red blood cell specific volume haematocrit; *ALT*, Alanine Aminotransferase; *TBIL*, total bilirubin; *Cr*, serum creatinine

### Univariate analysis of the occurrence of delirium in the training set

**Table 2:** Univariate analysis of the nondelirium group and delirium group in the training set (demographic characteristics)

Variable	Total	Non-delirium group	Delirium group	$\chi ^{2}$/t	*P*
	n (%)/${\bar{x}} \pm s$	n (%)/${\bar{x}} \pm s$	n (%)/${\bar{x}} \pm s$		
Gender				0.004	0.948
Male	232 (59.6)	119 (59.8)	113 (59.5)		
Female	157 (40.4)	80 (40.2)	77 (40.5)		
Age	59.8 ± 11.2	58.5 ± 12.3	61.1 ± 9.9	−2.296	0.022*
BMI	24.0 ± 3.4	24.1 ± 3.4	23.9 ± 3.4	0.638	0.524
Blood type				0.957	0.812
A	133 (34.2)	64 (32.2)	69 (36.3)		
B	107 (27.5)	55 (27.6)	52 (27.4)		
AB	33 (8.5)	17 (8.5)	16 (8.4)		
O	116 (29.8)	61 (31.7)	53 (27.9)		
Past history					
Hypertension	175 (45.0)	91 (45.7)	84 (44.2)	0.091	0.764
Diabetes	63 (16.2)	32 (16.1)	31 (16.3)	0.004	0.950
Stroke	23 (5.9)	15 (7.5)	8 (4.2)	1.934	0.164
Atrial fibrillation	92 (23.7)	46 (23.1)	46 (24.2)	0.065	0.799
Malignant tumor	15 (3.9)	7 (3.5)	8 (4.2)	0.126	0.723

*BMI*, body mass index.

*$$P<$$ 0.05

Age, type of surgery, CPB duration, intraoperative minimum MAP, intraoperative blood transfusion, postoperative MV, postoperative TBIL, postoperative serum urea nitrogen, postoperative Cr, postoperative serum sodium, and postoperative serum albumin were significantly different between the delirium and nondelirium groups ($P<$ 0.05) (Tables 2, 3, 4).

**Table 3:** Univariate analysis of the nondelirium group and delirium group in the training set (intraoperative factors)

Variable	Total	Non-delirium group	Delirium group	$\chi ^{2}$/t	*P*
	n(%)/${\bar{x}} \pm s$	n(%)/${\bar{x}} \pm s$	n(%)/${\bar{x}} \pm s$		
Type of surgery				17.479	0.004*
CABG	96 (24.7)	57 (28.6)	39 (20.5)		
Valve replacement or shaping	185 (47.6)	89 (44.7)	96 (50.5)		
Aortic valve replacement or shaping	18 (4.6)	5 (2.5)	13 (6.8)		
Heart tumor removal	10 (2.6)	10 (5.0)	0 (0.0)		
Congenital correction	23 (5.9)	12 (6.0)	11 (5.8)		
Two kinds of surgery and above	57 (14.7)	26 (13.1)	31 (16.3)		
CPB duration	98.8 ± 73.3	83.5 ± 65.7	114.8 ± 77.5	−4.292	<0.001*
Intraoperative minimum MAP	41.1 ± 11.2	42.8 ± 11.3	39.3 ± 10.8	3.138	0.002*
Intraoperative blood transfusion	72 (18.5)	26 (13.1)	46 (24.2)	8.005	0.005*

*CPB*, cardiopulmonary bypass; *MAP*, mean arterial pressure.

*$$P<$$ 0.05

**Table 4:** Univariate analysis of the nondelirium group and delirium group in the training set (postoperative factors)

Variable	Total	Non-delirium group	Delirium group	t	*P*
	n(%)/${\bar{x}} \pm s$	n(%)/${\bar{x}} \pm s$	n(%)/${\bar{x}} \pm s$		
Postoperative MV	33.3 ± 71.5	21.8 ± 18.1	45.2 ± 99.4	−3.193	0.002*
Postoperative WBC	13.4 ± 4.3	13.3 ± 4.0	13.6 ± 4.5	−0.714	0.475
Postoperative Hct	0.31 ± 0.04	0.31 ± 0.04	0.31 ± 0.04	1.780	0.076
Postoperative ALT	48.6 ± 68.9	43.5 ± 47.5	53.9 ± 85.6	−1.498	0.135
Postoperative TBIL	25.8 ± 14.4	23.9 ± 13.3	27.9 ± 15.3	−2.713	0.007*
Postoperative serum albumin	31.6 ± 5.9	32.3 ± 6.1	30.9 ± 5.7	2.201	0.028*
Postoperative serum urea nitrogen	9.2 ± 3.5	8.8 ± 3.4	9.7 ± 3.6	−2.680	0.008*
Postoperative Cr	101.8 ± 41.7	93.4 ± 34.3	110.6 ± 46.7	−4.106	<0.001*
Postoperative serum sodium	146.0 ± 4.3	145.0 ± 4.0	147.2 ± 4.3	−5.336	<0.001*

*MV*, mechanical ventilation; *WBC*, white blood cell; *Hct*, red blood cell specific volume haematocrit; *ALT*, alanine aminotransferase; *TBIL*, total bilirubin; *Cr*, serum creatinine

### Multivariate logistic regression of delirium in the training set

Logistic regression analysis showed that CPB duration (OR $=$ 1.004, 95% CI: 1.001–1.008, $P=$ 0.018), postoperative serum sodium (OR $=$ 1.112, 95% CI: 1.049–1.178, $P<$ 0.001), age (OR $=$ 1.027, 95% CI: 1.006–1.048, $P=$ 0.011), and postoperative MV (OR $=$ 1.019, 95% CI: 1.008–1.030, $P<$ 0.001) were independent risk factors for delirium, and the coefficient of determination $R^{2}$ was 0.200 (Table 5).

**Table 5:** Logistic regression analysis results of postoperative delirium in cardiovascular surgery patients

Variable	B	S.E.	Wald	OR	95% C.I.	*P*
CPB duration (minutes)	0.004	0.002	5.598	1.004	1.001–1.008	0.018
Postoperative serum sodium (mmol/L)	0.106	0.030	12.837	1.112	1.049–1.178	<0.001
Age (years old)	0.027	0.010	6.505	1.027	1.006–1.048	0.011
Postoperative MV (hours)	0.019	0.005	12.363	1.019	1.008–1.030	<0.001
Constant	−18.025	4.361	17.085			<0.001

*CPB*, cardiopulmonary bypass; *MV*, mechanical ventilation

### Predictive nomogram model for delirium in patients with cardiovascular surgery

**Fig. 1:**
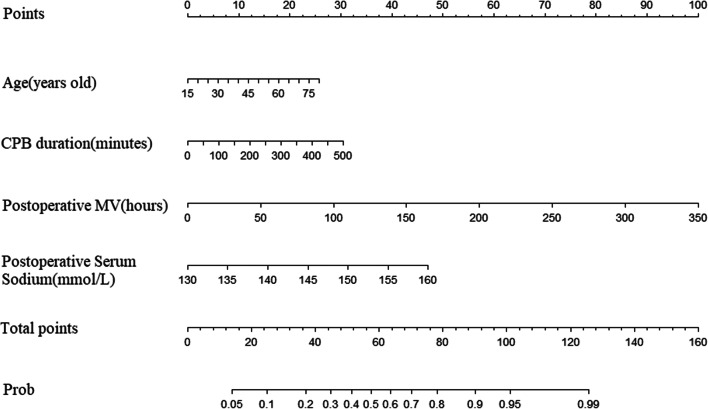
Prediction nomogram model for delirium in patients undergoing cardiovascular surgery

In this study, a nomogram model was constructed based on four independent risk factors: age, CPB duration, postoperative MV, and postoperative serum sodium (Fig. 1).

### Discrimination and calibration of the nomogram

**Fig. 2:**
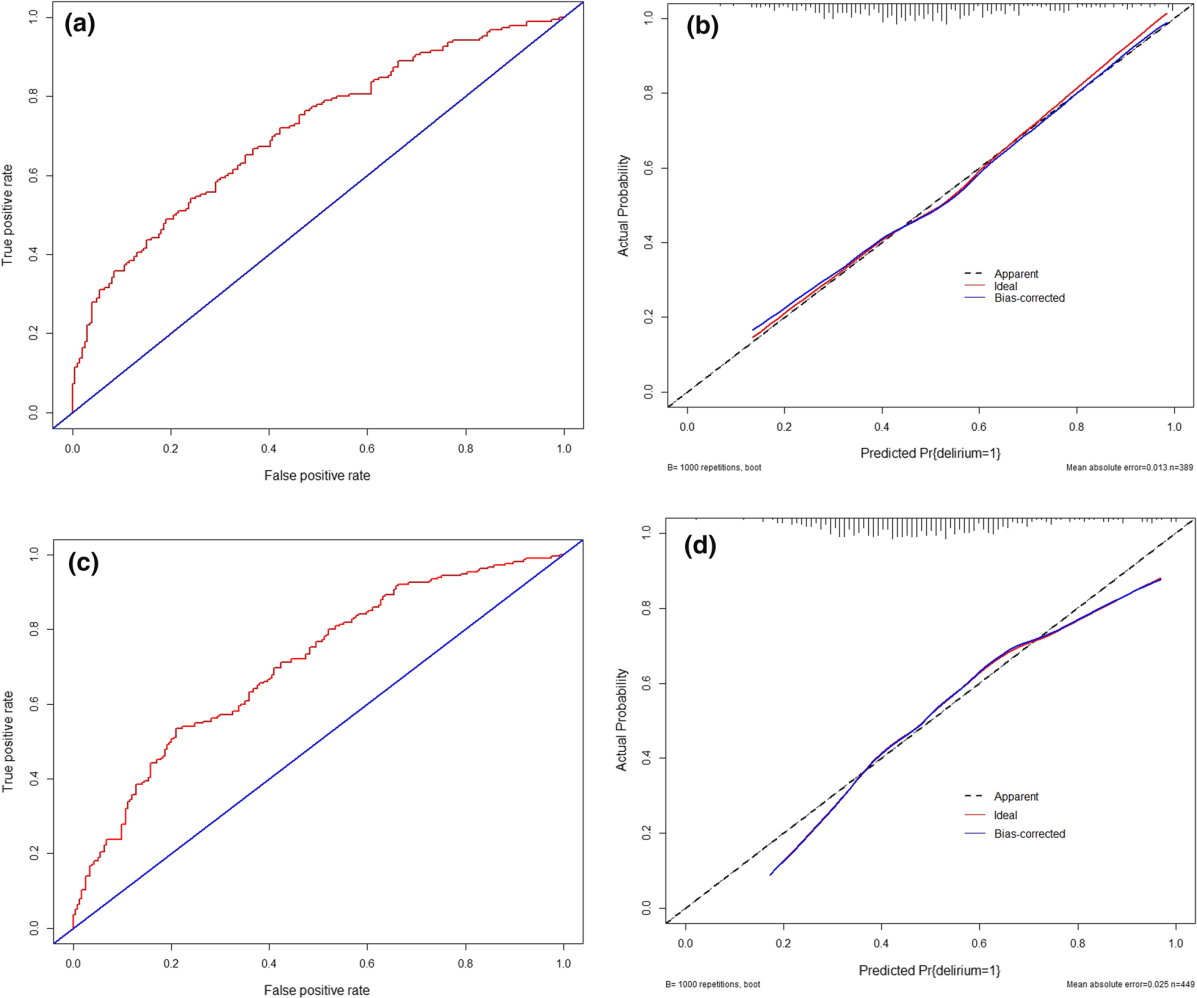
**a** ROC curves for the nomogram model in the training set; **b** Calibration curves for the nomogram model in the training set; **c** ROC curves for the nomogram model in the validation set; **d** Calibration curves for the nomogram model in the validation set

In the training set, AUC$^\text {ROC}$ was 0.712, and the 95% CI was 0.661–0.762. The results indicated that the model had a moderate discriminative ability (Fig. 2A). The goodness-of-fit test indicated that the fit was good ($\chi ^{2}=$ 6.200, $P=$ 0.625). The results showed that this model had acceptable goodness-of-fit and calibration. The calibration curve shows that the predicted incidence of the model was in good agreement with the actual incidence (Fig. 2B).

### Validation of the predictive accuracy of the nomogram

The established model was used in the validation set to further verify the predictive performance. The ROC curve in the verification set is shown in Fig. 2C. The area under the curve (AUC$^\text {ROC}$) was 0.705, the 95% CI was 0.657–0.752, and the calibration curve showed good agreement between the predicted incidence and the actual incidence (Fig. 2D). The goodness-of-fit test indicated that the fit was good ($\chi ^{2}=$ 8.6533, $P=$ 0.372). The result showed that this model had acceptable goodness-of-fit and calibration.

## Discussion

Postoperative delirium is associated with an increased number of different complications, such as prolonged hospitalisation, long-term cognitive impairment and increased mortality [14]. Fortunately, up to 40% of delirium cases can be prevented [1]. Therefore, strengthening the monitoring of delirium and supporting early prevention and identification will be an important part of the care of patients undergoing cardiovascular surgery. This study constructed a risk prediction model based on age, CPB duration, postoperative MV, and postoperative serum sodium for delirium in patients undergoing cardiovascular surgery and provided medical staff with an accurate and objective assessment tool.

In a previous study [15], a systematic review was conducted to identify three published high-quality intensive care unit delirium risk prediction models after cardiovascular surgery and assessed the transportability of the risk prediction models in cardiac surgery cohorts. Three published high-quality delirium risk prediction models were identified: Katznelson, the original PRE-DELIRIC, and the international recalibrated PRE-DELIRIC model. Finally, A. Lee et al. performed a logistic calibration on the international recalibrated PRE-DELIRIC model and found acceptable discrimination (0.75, 95% CI: 0.72–0.79) and good calibration. However, the international recalibrated PRE-DELIRIC model cannot be used to assess the risk of delirium within 24 h of ICU admission. The early delirium risk prediction model (E-PRE-DELIRIC) (0.70, 95% CI: 0.67–0.74, for delirium that developed < 2 days) constructed by Wassenaar [16] can complete delirium risk assessment when patients are admitted to the ICU. However, it has not been validated in a Chinese postcardiac surgery patient cohort. Domestic scholars [17] constructed a delirium prediction model with Chinese patients (an AUC of 0.819); their cohort, however, included only patients undergoing coronary artery bypass grafting. Moreover, the external validity of the scores presented by Liping T et al. have not been evaluated in other cardiac surgery cohorts.

Our model has a wider scope of application and can be used to assess the risk of delirium in all patients with cardiovascular surgery. The independent risk factors included in the prediction model of this study are easy to obtain, and the nomogram model is characterised by its ability to individually and intuitively express the results of a complex statistical model and assign corresponding scores to the influencing factors of the end-point event. Points are assigned to calculate the probability of an end-point event. This allows medical staff to start the assessment at the beginning of the patient’s postoperative CSICU treatment, and it is easier to use the iconic model for assessment, which is convenient for nursing staff.

This study found that age was an independent risk factor for the occurrence of delirium in patients after cardiovascular surgery. Numerous studies [2] have shown that advanced age is a high-risk factor for postoperative delirium. Elderly patients experience degeneration of bodily functions and brain tissue. They also have reduced levels of various central neurotransmitters, such as acetylcholine and epinephrine. At the same time, due to obstructed brain function, patients often have cognitive dysfunction, which may be the cause of the high incidence of delirium [18, 19]. CPB duration was significantly related to the occurrence of postoperative delirium. The longer the CPB duration was, the higher the incidence of delirium. This finding is similar to that of previous studies [20]. One of the hypotheses for the development of delirium is the development of systemic inflammatory response syndrome (SIRS) not only due to the cardiovascular surgery itself but also due to the exposure of the patient to the adverse effect of CPB [20, 21]. The main reason is the need to block the aorta during CPB and perform blood thinning. Microthrombosis will inevitably be caused during CPB. Harmful substances will also be produced after low-temperature circulation and rewarming. The above factors have an impact on cerebral blood perfusion, resulting in ischaemic and hypoxic changes in brain tissue. The results of this study showed that postoperative mechanical ventilation time is an independent risk factor for the occurrence of postoperative delirium. The longer the mechanical ventilation time is, the higher the incidence of delirium. In recent years, a large number of studies have shown that mechanical ventilation is a related risk factor for delirium [2]. While mechanical ventilation has therapeutic effects, it also changes the patient’s normal haemodynamics and respiratory physiology and is likely to cause extreme physical and psychological discomfort for the patient. In addition, mechanically ventilated patients are prone to sleep disorders. Patients who are mechanically ventilated have more fragmented and daytime sleep and reduced sleep efficiency than patients who are not mechanically ventilated [22]. In addition, the inclusion of sleep disturbance in the Diagnostic and Statistical Manual of Mental Disorders, 5th Edition in its constellation of symptoms used in diagnosing delirium has increased awareness of the link between sleep and delirium [22]. Patients undergoing cardiovascular surgery routinely require ventilator-assisted ventilation, which leads to a high incidence of postoperative delirium. In the off-line assessment process, the patient has delirium and cannot cooperate with the assessment, which makes it difficult to make accurate off-line decisions. At present, there is no uniform standard in academic circles for prolonged mechanical ventilation (PMV). PMV is generally defined as mechanical ventilation for more than 24 h in patients after cardiac surgery [23, 24]. Therefore, the inability to remove the ventilator within 24 h after cardiac surgery should strongly attract the attention of medical staff, and the relevant measures should be taken to shorten the time of mechanical ventilation. At present, the ABCDEF clustered delirium prevention strategy proposed by the 2017 National Critical Care Medicine Conference [25] and eCASH strategy [26] are most commonly used. The results of this study showed that postoperative hypernatraemia is a high-risk factor for postoperative delirium. Hypernatraemia refers to electrolyte disorders with a blood sodium concentration of > 145 mmol/L. A study by Hong Liang [27] showed that hypernatraemia increases the probability of postoperative delirium, which is consistent with our research results. Theologou et al. [28] also showed that the risk of developing delirium was associated with prolonged endotracheal intubation and prolonged ICU stay, along with increased urea, neutrophil-to-lymphocyte ratio, creatinine, and sodium levels. This finding might suggest that an inadequate reaction of the immune system may play a role in the pathogenesis of delirium [14]. Therefore, medical staff should correct the patients’ hypernatraemia in time and maintain a stable internal environment.

The nomogram model constructed in this study was used to score patients after cardiovascular surgery, and the risk of delirium was calculated for each patient to achieve individualized prediction. For example, if the patient is 70 years old, draw a vertical line from the point on the age scoring axis to the point axis, and the corresponding score for the patient is 22 points. The scores of other items were measured in the same way, and the CPB duration was 150 min (score $=$ 10 points), postoperative serum sodium was 145 mmol/L (score $=$ 25 points), and postoperative MV was 50 min (score $=$ 15 points). Then, the total score of the nomogram was $22+ 10 + 25 + 15 = 72$ points, and the corresponding risk of delirium was 0.72; that is, the patient had a 72% risk of developing delirium. As a patient’s risk becomes higher, medical staff should pay increased attention to him or her, and the patient should be actively intervened. When the patient cannot be taken off the ventilator within 24 h, the mechanical ventilation time is prolonged, or the serum sodium concentration changes, medical staff should dynamically evaluate the patient and take intervention measures to reduce injuries, reduce the risk of delirium and improve the prognosis.

### Limitation

There are some limitations of our study. Because of limited resources, we were unable to perform a multicentre study, and the calibration and discrimination were not tested in other medical centres.

## Conclusion

We established a new nomogram model that can provide an individualized prediction of delirium in cardiovascular surgical patients. This model may provide nurses with an accurate and objective assessment tool to identify high-risk delirium in cardiovascular surgical patients.

## Data Availability

The datasets generated and analysed during the current study are not publicly available due to the privacy of the patients and confidentiality of the Hospital information system but are available from the corresponding author on reasonable request.

## Abbreviations

CPB: Cardiopulmonary bypass
MV: Mechanical ventilation
AUC: Area under curve
ROC: Receiver-operating characteristic curve
ICU: Intensive care unit
CSICU: Cardiovascular surgery intensive care unit
MAP: Mean arterial pressure

## References

[1] Aldecoa C, Bettelli G, Bilotta F, Sanders RD, Audisio R, Borozdina A, Cherubini A, Jones C, Kehlet H, MacLullich A, Radtke F, Riese F, Slooter AJ, Veyckemans F, Kramer S, Neuner B, Weiss B, Spies CD (2017). European society of anaesthesiology evidence-based and consensus-based guideline on postoperative delirium. Eur J Anaesthesiol.

[2] Chen H, Mo L, Hu H, Ou Y, Luo J (2021). Risk factors of postoperative delirium after cardiac surgery: a meta-analysis. J Cardiothorac Surg.

[3] Hshieh TT, Inouye SK, Oh ES (2018). Delirium in the elderly. Psychiatr Clin North Am.

[4] Järvelä K, Porkkala H, Karlsson S, Martikainen T, Selander T, Bendel S (2018). Postoperative delirium in cardiac surgery patients. J Cardiothorac Vasc Anesth.

[5] Inouye SK, Westendorp RGJ, Saczynski JS (2014). Delirium in elderly people. Lancet.

[6] Al-Qadheeb NS, Balk EM, Fraser GL, Skrobik Y, Riker RR, Kress JP, Whitehead S, Devlin JW (2014). Randomized icu trials do not demonstrate an association between interventions that reduce delirium duration and short-term mortality: a systematic review and meta-analysis. Crit Care Med.

[7] Yu C, Zhang Y. Development and validation of prognostic nomogram for young patients with gastric cancer. Ann Transl Med 2019;7(22):641. 10.21037/atm.2019.10.77 10.21037/atm.2019.10.77 PMC6944578 31930042

[8] Ranstam J, Cook JA, Collins GS (2016). Clinical prediction models. Br J Surg.

[9] Damluji AA, Forman DE, van Diepen S, Alexander KP, Page n RL, Hummel SL, Menon V, Katz JN, Albert NM, Afilalo J, Cohen MG. Older adults in the cardiac intensive care unit: factoring geriatric syndromes in the management, prognosis, and process of care: a scientific statement from the american heart association. Circulation 2020;141(2):6–32. 10.1161/cir.0000000000000741 10.1161/CIR.0000000000000741 31813278

[10] Moons KG, Altman DG, Reitsma JB, Ioannidis JP, Macaskill P, Steyerberg EW, Vickers AJ, Ransohoff DF, Collins GS (2015). Transparent reporting of a multivariable prediction model for individual prognosis or diagnosis (tripod): explanation and elaboration. Ann Intern Med.

[11] Collins GS, Reitsma JB, Altman DG, Moons KG (2015). Transparent reporting of a multivariable prediction model for individual prognosis or diagnosis (tripod): the tripod statement. Bmj.

[12] Ely EW, Margolin R, Francis J, May L, Truman B, Dittus R, Speroff T, Gautam S, Bernard GR, Inouye SK (2001). Evaluation of delirium in critically ill patients: validation of the confusion assessment method for the intensive care unit (cam-icu). Crit Care Med.

[13] Chen Y, Du H, Wei BH, Chang XN, Dong CM (2017). Development and validation of risk-stratification delirium prediction model for critically ill patients: a prospective, observational, single-center study. Medicine (Baltimore).

[14] Majewski P, Zegan-Barańska M, Karolak I, Kaim K, Żukowski M, Kotfis K (2020). Current evidence regarding biomarkers used to aid postoperative delirium diagnosis in the field of cardiac surgery-review. Medicina.

[15] Lee A, Mu JL, Joynt GM, Chiu CH, Lai VKW, Gin T, Underwood MJ (2017). Risk prediction models for delirium in the intensive care unit after cardiac surgery: a systematic review and independent external validation. Br J Anaesth.

[16] Wassenaar A, van den Boogaard M, van Achterberg T, Slooter AJ, Kuiper MA, Hoogendoorn ME, Simons KS, Maseda E, Pinto N, Jones C, Luetz A, Schandl A, Verbrugghe W, Aitken LM, van Haren FM, Donders AR, Schoonhoven L, Pickkers P (2015). Multinational development and validation of an early prediction model for delirium in icu patients. Intensive Care Med.

[17] Liping T, Xia D, WangYi EA (2017). Research progress on delirium risk prediction models for patients after coronary artery bypass graft. J Nurs Sci.

[18] Oh ES, Li M, Fafowora TM, Inouye SK, Chen CH, Rosman LM, Lyketsos CG, Sieber FE, Puhan MA (2015). Preoperative risk factors for postoperative delirium following hip fracture repair: a systematic review. Int J Geriatr Psychiatry.

[19] Yoshitaka S, Egi M, Kanazawa T, Toda Y, Morita K (2014). The association of plasma gamma-aminobutyric acid concentration with postoperative delirium in critically ill patients. Crit Care Resusc.

[20] Berger M, Terrando N, Smith SK, Browndyke JN, Newman MF, Mathew JP (2018). Neurocognitive function after cardiac surgery: from phenotypes to mechanisms. Anesthesiology.

[21] Stachon P, Kaier K, Zirlik A, Reinöhl J, Heidt T, Bothe W, Hehn P, Zehender M, Bode C, von Zur Mühlen C (2018). Risk factors and outcome of postoperative delirium after transcatheter aortic valve replacement. Clin Res Cardiol.

[22] Pisani MA, D’Ambrosio C (2020). Sleep and delirium in adults who are critically ill: a contemporary review. Chest.

[23] Jin M, Ma WG, Liu S, Zhu J, Sun L, Lu J, Cheng W (2017). Predictors of prolonged mechanical ventilation in adults after acute type-a aortic dissection repair. J Cardiothorac Vasc Anesth.

[24] Fernandez-Zamora MD, Gordillo-Brenes A, Banderas-Bravo E, Arboleda-Sánchez JA, Hinojosa-Pérez R, Aguilar-Alonso E, Herruzo-Aviles A, Curiel-Balsera E, Sánchez-Rodríguez A, Rivera-Fernández R (2018). Prolonged mechanical ventilation as a predictor of mortality after cardiac surgery. Respir Care.

[25] Pun BT, Balas MC, Barnes-Daly MA, Thompson JL, Aldrich JM, Barr J, Byrum D, Carson SS, Devlin JW, Engel HJ, Esbrook CL, Hargett KD, Harmon L, Hielsberg C, Jackson JC, Kelly TL, Kumar V, Millner L, Morse A, Perme CS, Posa PJ, Puntillo KA, Schweickert WD, Stollings JL, Tan A, D’Agostino McGowan L, Ely EW (2019). Caring for critically ill patients with the abcdef bundle: results of the icu liberation collaborative in over 15,000 adults. Crit Care Med.

[26] Vincent JL, Shehabi Y, Walsh TS, Pandharipande PP, Ball JA, Spronk P, Longrois D, Strøm T, Conti G, Funk GC, Badenes R, Mantz J, Spies C, Takala J (2016). Comfort and patient-centred care without excessive sedation: the ecash concept. Intensive Care Med.

[27] Liang H, Xiao S, Chang S (2020). Hypernatremia increases the incidence of late delirium after cardiac surgery. Chin J Clin Thorac Cardiovasc Surg.

[28] Theologou S, Giakoumidakis K, Charitos C (2018). Perioperative predictors of delirium and incidence factors in adult patients post cardiac surgery. Pragmat Obs Res.

